# Metabolic control during the first two years of the COVID-19 pandemic in pediatric patients with type 1 diabetes: results from the German DPV initiative

**DOI:** 10.1007/s00592-023-02050-x

**Published:** 2023-03-04

**Authors:** Johanna Hammersen, Sascha R. Tittel, Semik Khodaverdi, Felix Reschke, Monika Flury, Ulrike Menzel, Kirsten Mönkemöller, Thomas Meissner, Beate Karges, Reinhard W. Holl

**Affiliations:** 1 Department of Pediatrics, University Hospital Erlangen, Loschgestr. 15, 91054 Erlangen, Germany; 2 Institute of Epidemiology and Medical Biometry, ZIBMT, University of Ulm, Ulm, Germany; 3 German Centre for Diabetes Research (DZD), Munich-Neuherberg, Germany; 4 Clinic for Children and Adolescent Medicine, Clinical Centre Hanau, Hanau, Germany; 5 Diabetes Center for Children and Adolescents, Children’s Hospital Auf Der Bult, Hannover, Germany; 6 Children’s Hospital Carl Gustav Carus, Technical University Dresden, Dresden, Germany; 7 Department of Paediatric Endocrinology, AKK Altonaer Kinderkrankenhaus, Hamburg, Germany; 8 Department of Pediatrics, Kinderkrankenhaus Amsterdamer Strasse, Cologne, Germany; 9 Department of General Pediatrics, Neonatology and Pediatric Cardiology, Medical Faculty, University Hospital, Duesseldorf, Germany; 10 Division of Endocrinology and Diabetology, Medical Faculty, RWTH Aachen University, Aachen, Germany; 11 Department of Pediatrics, Bethlehem Hospital Stolberg, Stolberg, Germany

**Keywords:** Metabolic control, SARS-CoV2 pandemic, Lockdown, Pediatric diabetes

## Abstract

**Aim:**

To assess effects of the SARS-CoV2 pandemic on metabolic control in youth with type 1 diabetes (T1D) in Germany in a population-based analysis.

**Methods:**

Data from 33,372 pediatric T1D patients from the Diabetes Prospective Follow-up (DPV) registry, with face-to-face visits or telemedicine contacts in the years 2019–2021, were available. Datasets from eight time periods between March 15, 2020, and December 31, 2021, according to SARS-CoV2 incidence waves, were compared to those from five control time periods. Parameters of metabolic control were assessed with adjustment for sex, age, diabetes duration, and repeated measurements. Laboratory-measured HbA1c values and those estimated from CGM were aggregated into a combined glucose indicator (CGI).

**Results:**

There was no clinically relevant difference in metabolic control between pandemic and control time periods with adjusted CGI values ranging from 7.61% [7.60–7.63] (mean [95% confidence interval (CI)]) in the third quarter of 2019 to 7.83% [7.82–7.85] in the time period from January 1 to March 15 2020, in the other control periods, and during the pandemic, CGI values lay between these values. BMI-SDS rose during the pandemic from 0.29 [0.28–0.30] (mean [95% CI]) in the third quarter of 2019 to 0.40 [0.39–0.41] during the fourth wave. Adjusted insulin dose rose during the pandemic. Event rates for hypoglycemic coma and diabetic ketoacidosis remained unchanged.

**Conclusions:**

We found no clinically relevant change of glycemic control or incidence of acute diabetes complications during the pandemic. The observed BMI increase may represent an important health risk for youth with T1D.

**Supplementary Information:**

The online version contains supplementary material available at 10.1007/s00592-023-02050-x.

## Introduction

For more than two years, the SARS-CoV2 pandemic has shaped daily life of children and their families as well as healthcare services and may have exerted a strong influence on diabetes management in pediatric patients with type 1 diabetes (T1D).

Depending on daily infection rates, there have been four waves of SARS-CoV2 pandemic in Germany in the years 2020 and 2021 [1]. In mid-March 2020, the first lockdown was implemented with nationwide closure of schools and daycare institutions, a recommendation for most employees to work in home-office, prohibition of team sport activities, and a closure of public gyms and playgrounds. After two months, in mid-May 2020, daily life was partially adapted back to normal. With the second SARS-CoV2 infection wave, social distancing means were gradually reimplemented in mid-October of 2020 and mostly retained until the end of the third infection wave in May 2021. Social distancing means were mostly suspended during the summer months of 2021, but reimplemented during the fourth wave in the fall of 2021. To guarantee education for all children, no statewide school closures were reimplemented in the second half of 2021. During the first months of the SARS-CoV2 pandemic in 2020, glycemic control in pediatric patients with T1D remained stable in different patient cohorts despite social distancing means [2–6]. An analysis of adult patients with T1D and T2D in Germany also shows stable metabolic control [7]. However, long-term effects of the ongoing pandemic may need to be evaluated.

This study aims at evaluating the effect of the ongoing COVID-19 pandemic on metabolic control in a large, nationwide cohort of pediatric T1D patients in Germany documented in the Diabetes Prospective Follow-up (DPV) registry, a multicenter quality improvement initiative for patients with diabetes covering more than 90% of youth with T1D in Germany.

## Methods

### Data source

Data originate from the DPV registry, in which participating German, Austrian, Swiss, and Luxembourgian diabetes treatment centers document data from diabetes-related visits for quality improvement and scientific research. Telemedicine contacts, hospitalizations, and outpatient visits are documented in the registry. Twice a year, pseudonymized data are transferred to the Institute of Epidemiology and Medical Biometry at Ulm University, where data are validated, anonymized, and aggregated into a cumulative registry.

### Study population

Data were retrieved from the DPV registry in August 2022.

**Fig. 1:**
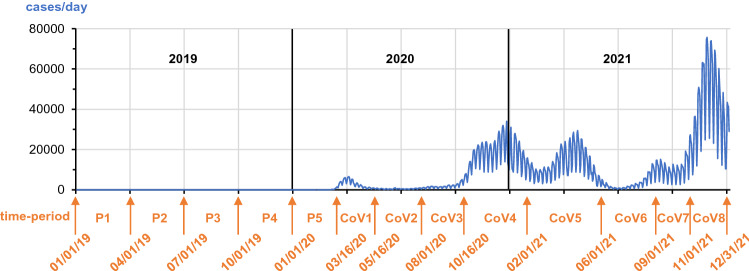
Duration of five pre-pandemic time periods (P1 – P5) as control and eight time periods during the pandemic (CoV1 – CoV8) is indicated, as well as the daily incidence of new SARS-CoV2 infections in Germany (blue graph)

Data were limited to patients with visits or telemedicine contacts in German diabetes centers in one or more defined time periods between January 1, 2019, and December 31, 2021. As a control, the year 2019 was divided into four quarters, time periods pre-pandemic (P) P1–P4. Time period P5, before the pandemic, ranges from January 1 to March 15, 2020. The remaining months of 2020 and the year 2021 were split into eight sections according to the SARS-CoV2 incidence waves, time periods CoV1–CoV8. To guarantee a similar length of the time periods, the phases of low incidence in the summer and fall of 2020 and 2021 are divided into two sections, respectively. Time period CoV1, the first wave, spans from March 16 to May 15, 2020. Time periods CoV2 and CoV3, May 16th–July 31st, 2020, and August 1st–October 15th, 2020, cover the phase of low incidence and decontainment in the summer and fall of 2020, time periods CoV4 and CoV5, October 16, 2020–January 31, 2021, and February 1–May 31, 2021, the second and third wave. The phase of low incidence and decontainment in the summer and fall of 2021 is represented by time periods CoV6 and CoV7, which lasted from June 1–August 31, 2021, and from September 1–October 31, 2021. Time period CoV8, November 1–December 31, 2021, covers the 4th wave of SARS-CoV2 incidence (Fig. 1).

**Fig. 2:**
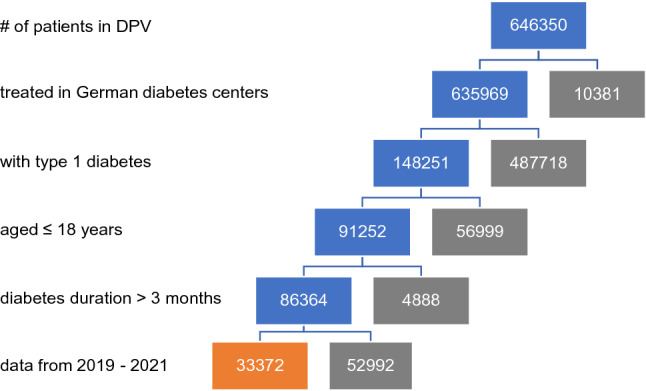
The process of patient selection is explained by showing the number of excluded patients in gray, the number of included patients in blue, and the number of patients in the final study population in orange

In total, 33,372 patients with T1D aged less than 18 years and with a diabetes duration longer than 3 months (to exclude data at onset) with visits in 274 German centers were included in the study (Fig. 2).

### Variables and outcomes

Documented clinical and demographic variables included sex, age at onset of diabetes, and age and duration of diabetes at each contact. A migratory background was assigned if the patient or at least one parent was born outside of Germany.^5^ Body weight standard deviation score (SDS), height SDS, and body mass index (BMI) SDS were based on the German Health Interview and Examination Survey for Children and Adolescents (KiGGs) reference [8].

HbA1c values were mathematically standardized to the reference range of 4.05–6.05% (IFCC 20.8–42.6 mmol/L) of the Diabetes Control and Complications Trial applying the multiple of the mean method in order to correct for different laboratory methods [5, 9]. Corrected HbA1c values were deducted from time in range (TiR) data as described previously [10]. Laboratory-measured HbA1c values and those estimated from CGM data were integrated into a combined glucose indicator (CGI) expressed in “%”, in analogy to HbA1c values as described before [5]. Acute complications—DKA and hypoglycemia—were also documented as outcomes. DKA was defined as presence of metabolic acidosis with a pH below 7.3 and/or bicarbonate levels below 15 mmol/L. Severe hypoglycemia was defined as an episode when the affected patient required assistance, hypoglycemic coma as loss of consciousness or seizure [11].

### Statistical analyses

For each patient, multiple data entries per time period were aggregated. In descriptive analyses, the median and the first and third quartiles were used for continuous variables. Sums were used to aggregate time of observation or the number of events for episodes of DKA, hypoglycemia, and hospitalization. For visits, face-to-face and telemedicine contacts were considered. The number of visits per patient and time interval were aggregated. The different lengths of the time intervals were standardized to one month (length of time interval in days multiplied by 365 and divided by 12).

For continuous outcomes, we calculated linear regression models. Event rates for DKA, hypoglycemia, hospitalization, and visits per month as well as CGM usage were estimated based on negative-binomial regression with the logarithm of individual time under risk as offset. All models were adjusted for the covariates sex, age (in groups of patients aged < 6 years, 6 to < 12 years, and 12–18 years), diabetes duration (in groups of patients with a T1D duration of ≤ 3 years, or > 3 years), and repeated measurements. A first-order autoregressive covariance structure was used to account for stronger correlation between closer measurements of an outcome. Models for daily insulin dose were additionally adjusted for usage of an insulin pump and BMI-SDS (in groups of patients with a BMI-SDS below 10th percentile, 10–90th percentile, or above 90th percentile). Outcomes are presented as adjusted least-square means with 95% confidence interval or as events per 100 patient-years with 95% confidence interval. Type III test of fixed effects for sex, age group, diabetes duration group, and time period were applied to the models. Trend tests were used for comparison of datasets before and during the pandemic. Two-sided *p*-values < 0.01 were considered significant.

For a sensitivity analysis, logistic regression models were additionally adjusted for the German Index of Socioeconomic Deprivation of 2012 (GISD_2012) as described before [12]. In an age-stratified analysis, models for CGI and BMI-SDS were analyzed in separate groups of patients aged < 12 years and ≥ 12 years.

All analyses were performed using SAS version 9.4, build TS1M7, on a windows server 2019 mainframe computer (Table 1).

**Table 1:** Unadjusted data for clinical characteristics, treatment and outcomes of patients with type 1 diabetes in different time periods. Median, 1st and 3rd quartiles are indicated for continuous variables, mean and percentages for categorical variables

Parameter	Time period												
	2019				2020					2021			
	P1	P2	P3	P4	P5	CoV1	CoV2	CoV3	CoV4	CoV5	CoV6	CoV7	CoV8
Dates	01/01/19–03/31/19	04/01/19–06/30/19	07/01/19–09/30/19	10/01/19–12/31/19	01/01/20–03/15/20	03/16/20–05/15/20	05/16/20–07/31/20	08/01/20–10/15/20	10/16/20–01/31/21	02/01/21–05/31/21	06/01/21–08/31/21	09/01/21–10/31/21	11/01/21–12/31/21
# of patients, total	20, 165	19, 893	19, 763	19, 853	18, 444	11, 990	17, 548	17, 731	20, 777	22, 466	20, 527	15, 577	15, 313
mean # of patients per month	6, 820	6, 654	6, 538	6, 568	7, 485	5, 983	6, 936	7, 101	5, 855	5, 698	6, 791	7, 772	7, 641
% male sex	52.5	52.7	52.5	52.7	52.7	52.6	52.6	52.5	52.8	52.6	52.6	52.3	53.2
% migratory background	25.0	25.0	25.0	25.5	25.9	26.9	26.3	26.5	26.2	26.5	26.4	27.2	27.0
age (years)	12.99 [9.79; 15.50]	12.99 [9.81; 15.51]	12.98 [9.77; 15.49]	13.06 [9.81; 15.53]	13.04 [9.80; 15.56]	13.10 [9.82; 15.61]	13.14 [9.90; 15.68]	13.04 [9.78; 15.58]	13.11 [9.84; 15.66]	13.10 [9.78; 15.69]	13.03 [9.71; 15.62]	12.99 [9.59; 15.53]	13.17 [9.81; 15.67]
diabetes duration (years)	4.34 [2.12; 7.44]	4.35 [2.12; 7.44]	4.38 [2.06; 7.51]	4.35 [2.08; 7.52]	4.33 [2.06; 7.54]	4.27 [2.03; 7.46]	4.42 [2.11; 7.60]	4.36 [2.00; 7.54]	4.33 [1.98; 7.61]	4.32 [1.97; 7.57]	4.29 [1.88; 7.51]	4.21 [1.88; 7.51]	4.27 [1.92; 7.56]
% insulin pump	58.8	59.2	59.9	59.8	60.3	59.0	60.1	60.2	60.3	61.0	61.6	61.8	61.8
% CGM usage	61.0	64.8	68.4	71.5	74.6	70.2	76.0	76.1	79.9	81.7	81.5	79.6	81.3
HbA1c (%)	7.52 [6.85; 8.30]	7.47 [6.78; 8.28]	7.45 [6.77; 8.23]	7.53 [6.86; 8.31]	7.68 [6.98; 8.48]	7.62 [6.92; 8.46]	7.47 [6.77; 8.28]	7.47 [6.80; 8.28]	7.55 [6.87; 8.31]	7.37 [6.73; 8.13]	7.41 [6.76; 8.19]	7.47 [6.77; 8.22]	7.48 [6.80; 8.28]
*N* available	19, 306	18, 987	18, 769	18, 872	17, 355	8, 708	15, 884	16, 020	18, 844	20, 783	18, 717	13, 457	13, 289
Proportion *N* available/# of patients (%)	95.74	95.44	94.97	95.06	94.10	72.63	90.52	90.35	90.70	92.51	83.31	86.39	86.78
TiR (%)	50 [38; 63]	50 [38; 62]	50 [39; 63]	49 [38; 62]	51[40; 64]	54 [41; 67]	54 [42; 67]	54 [42; 67]	54 [42; 67]	56 [43; 69]	56 [43; 68]	55 [43; 67]	54 [41; 67]
*N* available	1, 617	1, 658	1, 944	2, 438	2, 903	2, 679	3, 842	4, 482	6, 271	8, 197	7, 931	6, 468	6, 484
CGI (%)	7.55 [6.87; 8.38]	7.52 [6.83; 8.31]	7.48 [6.80; 8.29]	7.58 [6.87; 8.38]	7.72 [7.01; 8.51]	7.68 [6.93; 8.55]	7.55 [6.83; 8.38]	7.58 [6.86; 8.38]	7.62 [6.89; 8.42]	7.48 [6.77; 8.28]	7.52 [6.80; 8.35]	7.58 [6.86; 8.41]	7.63 [6.87; 8.48]
*N* available	19, 444	19, 163	18, 973	19, 194	17, 734	10, 108	16, 671	16, 821	19, 772	21, 835	19, 817	14, 806	14, 495

## Results

### Description of the study population

A total of 33,372 patients were included in the study (Fig. 2). The absolute number of patients in the time periods P1–P5 and CoV1–CoV8 varied from 11,990 in time period CoV1 to 22,466 in time period CoV5 (table). In the different time periods, the number of patients per month ranged from 5698 in time period CoV5 to 7772 in time period CoV7. The number of patients per month was lower during the first, second and third SARS-CoV2 incidence than in the year 2019, but increased to pre-pandemic values thereafter (table). When data from the most recent time period for each patient were considered, individuals in the whole cohort of patients had a median age of 14.3 years [1st and 3rd quartile: 10.7; 17.1], and the median duration of T1D was 4.9 years [2.3; 8.4]. 53.3% of the patients in the cohort were male, 25.7% had a migratory background, 58.1% administered insulin with an insulin pump, and 75.3% of the patients used CGM. Demographic differences between the groups were minimal (table).

Based on previous entries in the DPV registry, the expected number of patients aged < 16 years and with a diabetes duration of > 3 months at the end of the year was calculated for the years 2019, 2020, and 2021. To assess how many pediatric T1D patients missed their follow-up visits before and during the pandemic, we compared the percentage of expected and documented patients aged < 16 years and with a diabetes duration of > 3 months in the years 2019, 2020, and 2020. In 2019, 20,072 of 22,566 expected patients (88.94%) had a documented visit in the DPV registry; for 2020 and 2021, the fraction of documented and expected patients were similar, 20,192 of 22,830 patients (88.44%) in 2020, and 21,195 of 23,540 (90.04%).

### Parameters of outcome and treatment during the pandemic compared to the preceding year

Parameters of treatment and outcome in the eight time periods during the pandemic and the five control time periods were analyzed. Based on regression models adjusted for sex, age, diabetes duration, and repeated measurements, CGI values ranged from 7.61% [7.60–7.63] (mean [95% confidence interval (CI)]) in the third quarter of 2019, time period P3, to 7.83% [7.82–7.85] in time period P5, in the other control periods and during the pandemic, CGI values lay between these values (figure S1).

In the year 2019, the adjusted BMI-SDS was the lowest in the third quarter, 0.29 [0.28–0.30], and the highest in the fourth quarter, time period P4, 0.33 [0.31–0.34]. During the two years of the pandemic 2020 and 2021, an increase in adjusted BMI-SDS up to 0.40 [0.39–0.41] in time period CoV8 was observed (figure S1; *p* < 0.0001).

Adjusted event rates for acute complications—diabetic ketoacidosis, severe hypoglycemia, and hypoglycemic coma—were also analyzed. Event rates for diabetic ketoacidosis before and during the pandemic did not differ significantly (*p* = 0.911). In all time periods, the mean rates of diabetic ketoacidosis lay between 1.56 [1.25–1.95] and 2.20 [1.72–2.82] events per 100 patient-years in time periods P5, and CoV1 respectively. With one exception—time period CoV6—adjusted mean severe hypoglycemia rates during all time periods before and during the pandemic were similar and ranged from 5.72 [4.84–6.75] events per 100 patient-years in time period CoV4 to 9.71 [8.30–11.36] events per 100 patient-years in time period P4. The highest severe hypoglycemia rate was estimated for time period CoV6 with 13.12 [11.29–15.24] events per 100 patient-years. Event rates for hypoglycemic coma before and during the pandemic did not differ significantly (*p* = 0.776) (figure S1).

Variables of treatment included daily insulin dose, CGM usage, and rates of visits and hospitalizations. In 2019, the adjusted insulin dose ranged from 0.833 [0.828–0.837] (median [95% CI]) units per kg body weight per day in time period P2 to 0.838 [0.833–0.843] in time period P4. During the pandemic, the adjusted insulin dose continuously rose to 0.882 [0.877–0.887] units per kg body weight per day in time period CoV5 and remained elevated until time period CoV8, when it was 0.878 [0.873–0.883] units per kg body weight per day (*p* < 0.0001).

With one exception—time period CoV6, there were less hospitalizations during the pandemic compared to the control periods. Before the pandemic, the mean rate of hospitalizations varied between 36.33 [34.65–38.10] per 100 patient-years in time period P5, and 44.49 [42.70–46.36] events per 100 patient-years in time period P3. The lowest hospitalization rate was observed in time period CoV1, when it dropped to 25.24 [23.42–27.22] events per 100 patient-years. In the following time periods, the corrected hospitalization rates remained lower than before the pandemic with one exception, time period CoV6 (figure S1).

The number of visits per month was variable before and during the pandemic: In 2019, the adjusted number of visits per month—face-to-face and telemedicine—ranged from 0.44 [0.43–0.44] visits per month in time period P4 to 0.48 [0.47–0.49] visits per month in time period P1. In time period P5, the adjusted number of visits per month rose to 0.52 [0.51–0.52]. During the pandemic, the number of visits varied between 0.39 [0.38–0.39] visits per month in time period CoV5, the third wave, and 0.57 [0.55–0.58] visits per month in time period CoV1 (figure S1). Thus, the adjusted number of visits per month in all control time periods lay between the highest and the lowest number of visits per month, which were both observed during high incidence waves.

CGM usage rose before and during the pandemic: In the first time period of 2019, patients used CGM for an adjusted mean of 189.2 [184.6–193.9] days per patient-year, in time period CoV8, for 259.9 [251.6–266.3] days per patient-year. This continuous increase in CGM usage was only interrupted in time period CoV1, the first lockdown, when CGM usage was less frequent than in the previous time period.

To account for the impact of increased CGM usage on CGI outcomes, we have analyzed adjusted laboratory-measured values before and during the pandemic for patients who do not use CGM, and for the subgroup of patients for whom HbA1c values based on time in range were not available in the respective time period. HbA1c trends before and throughout the pandemic were similar to those observed for CGI values in the whole cohort. However, patients who do not use CGM at all had slightly higher estimated HbA1c values than those with no available TiR (figure S4).

**Fig. 3:**
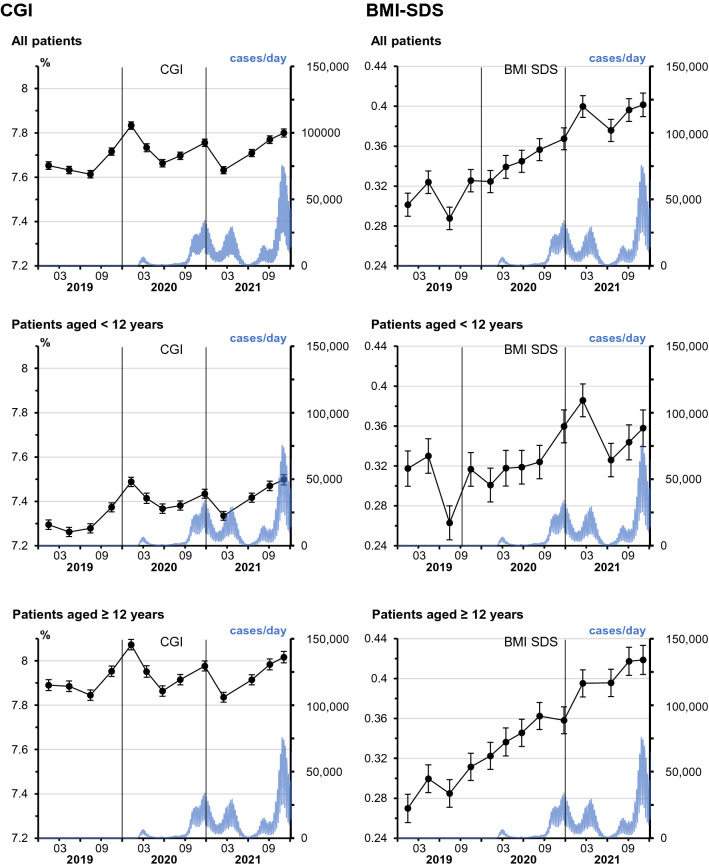
Estimated means and 95% confidence intervals are depicted for CGI (left column) and for BMI-SDS (right column) for all patients (upper row), for patients aged < 12 years (middle row), and for those aged ≥ 12 years (lower row). Models were corrected for differences of age, sex, diabetes duration, and for multiple measurements

In an additional age-stratified analysis, models for CGI and BMI-SDS were analyzed in separate groups of patients aged < 12 years and ≥ 12 years. A significant rise of BMI-SDS during the pandemic compared to before the pandemic was observed for children aged ≥ 12 years. In those aged < 12 years, changes of BMI-SDS were not significant. CGI values in children < 12 years were lower than in older children and varied between 7.26% [7.24–7.28] in time period P2, and 7.50% [7.48–7.52] in time period CoV8; in patients aged ≥ 12 years, adjusted CGI values lay between 7.85% [7.82–7.87] in time period P3 and 8.07% [8.05–8.10] in time period P5. In both age groups, there was no significant difference of CGI values before and during the pandemic (Fig. 3).

In a sensitivity analysis, regression analyses for CGI and BMI-SDS were additionally adjusted for social deprivation according to the GISD_2012. The patients were divided into terciles according to their social deprivation index, with 32.5, 32.2, and 35.3% of the patients being in the least deprived, medium deprived and most deprived tercile, respectively. The results of the logistic regression models in the sensitivity analyses were similar (supplementary material). Since a language barrier may pose an obstacle for remote visits [13, 14], a stratified analysis of the number of visits per month was performed for patients with and without migratory background. In all time periods before and during the pandemic, patients with a migratory background had a slightly higher frequency of visits than patients without migratory background (supplementary material).

## Discussion

In our cohort of 33,372 pediatric patients with T1D treated in Germany, in the years 2020 and 2021, no clinically relevant change of glycemic control, and no relevant change in the incidence of acute complications of T1D—DKA episodes and hypoglycemic coma—was observed.

Previous studies have shown that metabolic control in our [5] and other cohorts of pediatric patients with T1D remained mostly stable or even improved slightly [15–21] during the first months of the SARS-CoV2 pandemic or during the entire year 2020.

Positive and negative effects of the pandemic may have been in balance, so that metabolic control remained stable. During the school closures, younger patients may have benefitted from an increase in time spent at home under parental guidance. For adolescents, the pandemic restrictions may have contributed to more stability in glycemic control. On the other hand, for parents and younger children, the pandemic may have increased stress, social isolation, and anxiety [22] with potential negative effects on diabetes management and metabolic control.

Whereas the incidence rates of hypoglycemic coma did not differ during and before the pandemic, severe hypoglycemic events occurred more often during one time period, CoV6, the phase of decontainment lasting from June 1 to August 31, 2021. The sudden changes in the patient's lifestyle—the transition from the acute phase of lockdown to relaxation and increased activity—potentially explain the observed increase of severe hypoglycemia.

During the pandemic, BMI-SDS increased in our cohort. An increase of BMI-SDS has also been observed in the general population [23–26], and in other cohorts of pediatric T1D patients [3, 27]. In our cohort and in a cohort of Portuguese children with T1D [3], BMI-SDS especially increased in adolescents aged ≥ 12 years. The difference between adolescents and younger children may be explained by higher sedentary time in the older age group as a consequence of home schooling. Younger children with T1D may have been supervised by their parents more closely even before the pandemic, so that differences of BMI-SDS were not as obvious as in children aged ≥ 12 years.

During the pandemic, an increase in screen time and sedentary behavior as well as a decrease in physical activity in children has been observed in previous analyses [28–31]. Of note, screen time before the pandemic negatively correlated with physical activity during phases of social isolation in German youth [32]. Sedentary behavior may negatively influence metabolic control in youth with T1D [33]. Moreover, disordered eating behavior of youth with T1D increased during the pandemic [34]. A decrease in physical activity and altered nutrition possibly caused the observed weight gain in our cohort. Diabetes care teams should encourage patients to stay physically active and should counsel them regarding their eating behavior and their screen time habits. This may—however—not be sufficient to counteract the increase in sedentary behavior; more systematic programs fostering physical activity in children’s and adolescents’ daily life may be necessary [35–38]. In this study, we compared BMI during the pandemic with the year 2019, and therefore, longer-term pre-pandemic trends in weight gain cannot be excluded.

Apparently, patients and their families were well able to maintain stable glycemic control during the pandemic. Besides clinical face-to-face visits, telemedicine contacts and the possibility of remote assistance by diabetes care teams may also have contributed to stability of metabolic control. The increase in CGM usage during the pandemic may have been an important precondition for the observed stability of glycemic control in phases of social isolation with reduced clinic visits [16, 39, 40].

During the pandemic, telemedicine usage for routine diabetes care rapidly increased in different countries [40], which may have caused difficulties in obtaining optimal care for some patients. Besides limited availability of electronic devices, a language barrier may pose an obstacle for remote visits, as observed in Spanish-speaking families in the USA [13, 14]. This was—however—not observed in our cohort, where patients with a migratory background had a slightly higher number of visits before and throughout the pandemic. Apparently, patients with a migratory background and diabetes care teams rapidly adapted to phases of social isolation. It has been observed before that patients with language barriers rapidly adapted to telemedicine visits once they had experience with video consultations [13]. To guarantee optimal care during phases of social isolation, patients for whom a very low number of visits are recognized should be convinced to visit their diabetes care team in future lockdown situations.

Our study has several limitations. Certainly, there is a pre-selection of patients who visited diabetes teams in the time of very strict contact restrictions. Especially patients who lost contact with their diabetes teams may have had worse glycemic control than those who remained in regular care. However, demographic data did not differ relevantly between patients seen in the various time segments, and the proportion of documented and expected patients in the DPV registry in the years 2019, 2020, and 2021 was similar. We included HbA1c estimates based on TiR measurements to reduce a potential bias by excluding patients with telemedicine contacts only.

A strength of our study is the conception as a population-based study with a very high number of pediatric patients with T1D from Germany. Moreover, rather than including a subgroup of patients in a clinical study, our analyses are based on real-world data derived from documented routine care, which also makes our study robust and significant. Most of the previous analyses of metabolic control in youth with T1D during the SARS-CoV2 pandemic cover only the first months after onset of the pandemic, whereas this analysis includes data of the first two years of the pandemic.

In summary, data from a population-based cohort of 33,372 pediatric patients with T1D treated in Germany show no clinically relevant change of glycemic control, namely no relevant increase of CGI values, and no relevant increase in acute complications of T1D during the first two years of the pandemic compared to the preceding year 2019. However, the observed increase in BMI-SDS may represent an important health risk, especially for adolescents with T1D, so strategies to increase physical activity even under pandemic conditions and teaching healthy eating habits need to be strengthened.

## Supplementary Information

Below is the link to the electronic supplementary material.

Supplementary file1 (PDF 101 KB)

Supplementary file2 (PDF 197 KB)

## Data Availability

To protect patient privacy, patient-level data cannot be shared with outside investigators. However, upon request and after agreement from the DPV scientific board, joint research projects are possible.

## References

[1] Robert-Koch-Institut Corona-Dashboard. https://experience.arcgis.com/experience/478220a4c454480e823b17327b2bf1d4. Accessed Sep 4 2022

[2] Danne T, Lanzinger S, de Bock M (2021). A worldwide perspective on COVID-19 and diabetes management in 22,820 children from the SWEET project: diabetic ketoacidosis rates increase and glycemic control is maintained. Diabetes Technol Ther.

[3] Duarte V, Mota B, Ferreira S, Costa C, Correia CC (2022). Impact of COVID-19 lockdown on glycemic control in type 1 diabetes. Arch Pediatr.

[4] Hakonen E, Varimo T, Tuomaala AK, Miettinen PJ, Pulkkinen MA (2022). The effect of COVID-19 lockdown on the glycemic control of children with type 1 diabetes. BMC Pediatr.

[5] Hammersen J, Reschke F, Tittel SR (2022). Metabolic control during the SARS-CoV-2 lockdown in a large German cohort of pediatric patients with type 1 diabetes: results from the DPV initiative. Pediatr Diabetes.

[6] Zechmann S, Hotz L, Di Gangi S, Baumgartl K, Plate A, Potlukova E (2021). Impact of SARS-CoV-2 lockdown on glycaemic control: a retrospective observational cohort study in a tertiary setting. J Clin Med.

[7] Hartmann B, Tittel SR, Femerling M (2022). COVID-19 lockdown periods in 2020: good maintenance of metabolic control in adults with Type 1 and Type 2 diabetes. Exp Clin Endocrinol Diabetes.

[8] Rosario AS, Kurth BM, Stolzenberg H, Ellert U, Neuhauser H (2010). Body mass index percentiles for children and adolescents in Germany based on a nationally representative sample (KiGGS 2003–2006). Eur J Clin Nutr.

[9] Rosenbauer J, Dost A, Karges B, Hungele A, Stahl A, Bächle C (2012). Improved metabolic control in children and adolescents with type 1 diabetes: a trend analysis using prospective multicenter data from Germany and Austria. Diabetes Care.

[10] Vigersky RA, McMahon C (2019). The relationship of hemoglobin A1C to time-in-range in patients with diabetes. Diabetes Technol Ther.

[11] Abraham MB, Jones TW, Naranjo D, Karges B, Oduwole A, Tauschmann M, Maahs DM (2018). ISPAD Clinical Practice Consensus Guidelines 2018: Assessment and management of hypoglycemia in children and adolescents with diabetes. Pediatr Diabetes.

[12] Hammersen J, Tittel SR, Warncke K (2021). Previous diabetic ketoacidosis as a risk factor for recurrence in a large prospective contemporary pediatric cohort: results from the DPV initiative. Pediatr Diabetes.

[13] Hsueh L, Huang J, Millman AK, Gopalan A, Parikh RK, Teran S, Reed ME (2021). Disparities in use of video telemedicine among patients with limited english proficiency during the COVID-19 pandemic. JAMA Netw Open.

[14] Samuels-Kalow ME, Chary AN, Ciccolo G (2022). Barriers and facilitators to pediatric telehealth use in English- and Spanish-speaking families: a qualitative study. J Telemed Telecare.

[15] Brener A, Mazor-Aronovitch K, Rachmiel M (2020). Lessons learned from the continuous glucose monitoring metrics in pediatric patients with type 1 diabetes under COVID-19 lockdown. Acta Diabetol.

[16] Choudhary A, Adhikari S, White PC (2022). Impact of the COVID-19 pandemic on manage-ment of children and adolescents with Type 1 diabetes. BMC Pediatr.

[17] Christoforidis A, Kavoura E, Nemtsa A, Pappa K, Dimitriadou M (2020). Coronavirus lock-down effect on type 1 diabetes management οn children wearing insulin pump equipped with continuous glucose monitoring system. Diabetes Res Clin Pract.

[18] Di Dalmazi G, Maltoni G, Bongiorno C (2020). Comparison of the effects of lockdown due to COVID-19 on glucose patterns among children, adolescents, and adults with type 1 diabetes: CGM study. BMJ Open Diabetes Res Care.

[19] Kofoed PE, Timm S (2022). The impact of COVID-19 lockdown on glycaemic control and use of health services among children followed at a Danish diabetes clinic. Acta Paediatr.

[20] Lombardo F, Salzano G, Bombaci B, Basile P, Lucania G, Alibrandi A, Passanisi S (2021). Has COVID-19 lockdown improved glycaemic control in pediatric patients with type 1 diabetes? An analysis of continuous glucose monitoring metrics. Diabetes Res Clin Pract.

[21] Schiaffini R, Barbetti F, Rapini N (2020). School and pre-school children with type 1 diabetes during Covid-19 quarantine: The synergic effect of parental care and technology. Diabetes Res Clin Pract.

[22] Wade C, Burton ET, Akinseye L, Nelson G, Smith-Young J, Kim A (2022). Increased anxiety symptoms in pediatric type 1 diabetes during the acute phase of COVID-19 lockdown. J Pediatr Endocrinol Metab.

[23] Jarnig G, Jaunig J, Kerbl R, Strenger V, Haeusler G, van Poppel MNM (2022). Acceleration in BMI gain following COVID-19 restrictions. A longitudinal study with 7- to 10-year-old primary school children. Pediatr Obes.

[24] Shalitin S, Phillip M, Yackobovitch-Gavan M (2022). Changes in body mass index in children and adolescents in Israel during the COVID-19 pandemic. Int J Obes (Lond).

[25] Vogel M, Geserick M, Gausche R (2022). Age- and weight group-specific weight gain patterns in children and adolescents during the 15 years before and during the COVID-19 pandemic. Int J Obes (Lond).

[26] Woolford SJ, Sidell M, Li X, Else V, Young DR, Resnicow K, Koebnick C (2021). Changes in body mass index among children and adolescents during the COVID-19 pandemic. JAMA.

[27] Cheng HP, Wong JSL, Selveindran NM, Hong JYH (2021). Impact of COVID-19 lockdown on glycaemic control and lifestyle changes in children and adolescents with type 1 and type 2 diabetes mellitus. Endocrine.

[28] Ganzar LA, Salvo D, Burford K, Zhang Y, Kohl HW, Hoelscher DM (2022). Longitudinal changes in objectively-measured physical activity and sedentary time among school-age children in Central Texas, US during the COVID-19 pandemic. Int J Behav Nutr Phys Act.

[29] Kharel M, Sakamoto JL, Carandang RR (2022). Impact of COVID-19 pandemic lockdown on movement behaviours of children and adolescents: a systematic review. BMJ Glob Health.

[30] Neville RD, Lakes KD, Hopkins WG, Tarantino G, Draper CE, Beck R, Madigan S (2022). Global changes in child and adolescent physical activity during the COVID-19 pandemic: a systematic review and meta-analysis. JAMA Pediatr.

[31] Viner R, Russell S, Saulle R (2022). School closures during social lockdown and mental health, health behaviors, and well-being among children and adolescents during the first COVID-19 wave: a systematic review. JAMA Pediatr.

[32] Wunsch K, Nigg C, Niessner C (2021). The impact of COVID-19 on the interrelation of physical activity, screen time and health-related quality of life in children and adolescents in Germany: results of the Motorik-modul study. Children (Basel).

[33] MacMillan F, Kirk A, Mutrie N, Matthews L, Robertson K, Saunders DH (2014). A systematic review of physical activity and sedentary behavior intervention studies in youth with type 1 diabetes: study characteristics, intervention design, and efficacy. Pediatr Diabetes.

[34] Gillon-Keren M, Propper-Lewinsohn T, David M, Liberman A, Phillip M, Oron T (2022). Exacerbation of disordered eating behaviors in adolescents with type 1 diabetes during the COVID-19 pandemic. Acta Diabetol.

[35] Giblin S, Scully P, Evers J (2022). Physical activity surveillance in adolescents with Type 1 diabetes: a pilot mixed-methods investigation. J Diabetes Res.

[36] Gilbert AS, Schmidt L, Beck A, Kepper MM, Mazzucca S, Eyler A (2021). Associations of physical activity and sedentary behaviors with child mental well-being during the COVID-19 pandemic. BMC Public Health.

[37] Quirk H, Blake H, Dee B, Glazebrook C (2015). "Having diabetes shouldn't stop them": healthcare professionals' perceptions of physical activity in children with Type 1 diabetes. BMC Pediatr.

[38] Béghin L, Thivel D, Baudelet JB, Deschamps T, Ovigneur H, Vanhelst J (2022). Change in physical fitness due to the COVID-19 pandemic lockdown in French adolescents: a comparison between two independent large samples from Diagnoform battery. Eur J Pediatr.

[39] Bassi G, Mancinelli E, Dell’Arciprete G, Salcuni S (2021). The impact of the Covid-19 pandemic on the well-being and diabetes management of adolescents with Type 1 diabetes and their caregivers: a scoping review. Int J Environ Res Public Health.

[40] Giani E, Dovc K, Dos Santos TJ (2021). Telemedicine and COVID-19 pandemic: The perfect storm to mark a change in diabetes care. Results from a world-wide cross-sectional web-based survey. Pediatr Diabetes.

