# Supplementary material for: Metabolic control during the first two years of the COVID-19 pandemic in pediatric patients with type 1 diabetes: results from the German DPV initiative
Source: Acta Diabetol. 2023 Mar 4;60(6):757–66. doi: 10.1007/s00592-023-02050-x (PMC9985474; doi:10.1007/s00592-023-02050-x)
Supplement: Supplementary file 1 — Supplementary file1 (PDF 101 KB) [file 592_2023_2050_MOESM1_ESM.pdf]

**Supplementary material**

**Figure S1**

Parameters of outcome and treatment in the time-periods of 2019, 2020, and 2021 (left y-axis), and SARS-CoV2 incidence waves (right y-axis, blue lines). Estimated means and 95% confidence intervals are depicted for parameters of outcome, CGI, BMI-SDS, severe hypoglycemia rates, hypoglycemic coma, and DKA rates, and for parameters of treatment, insulin dose, hospitalizations, and visits. Models were corrected for differences of age, sex, diabetes duration, and multiple measurements, those for insulin dose additionally for usage of insulin pumps and BMI-SDS.

**Figure S2**

Estimated means and 95% confidence intervals are depicted for CGI (left column) and for BMI-SDS (right column) for all patients (upper row), for patients aged < 12 years (middle row), and for those aged ≥12 years (lower row). Models were corrected for differences of age, sex, diabetes duration, multiple measurements, and for social deprivation according to the GISD_2012.

**Figure S3**

Estimated means and 95% confidence intervals are depicted for a stratified analysis of the number of visits per month for patients with migratory background (black), and without migratory background (grey). Models were adjusted for differences of age, sex, diabetes duration, and multiple measurements.

**Figure S4**

Estimated means and 95% confidence intervals are depicted for laboratory-measured HbA1c for patients not using CGM (left panel) and for patients with no available TiR data in the respective time-period (right panel). Models were corrected for differences of age, sex, diabetes duration, and multiple measurements.

**Participating centers**

Aachen - Uni-Kinderklinik RWTH, Aalen Kinderklinik, Ahlen St. Franziskus Kinderklinik, Altötting Kinderklinik Zentrum Inn-Salzach, Amberg Kinderklinik St. Marien, Arnsberg-Hüsten Karolinenhosp. Kinderabteilung, Aue Helios Kinderklink Augsburg Josefinum Kinderklinik, Augsburg Uni-Kinderklinik, Aurich Kinderklinik, Bad Aibling Internist. Praxis, Bad Hersfeld Kinderklinik, Bad Kreuznach Diakonie Kikli, Bad Kreuznach-Viktoriastift, Bad Kösen Median Kinderklinik, Bad Mergentheim – Diabetesfachklinik, Bad Mergentheim - Kinderdiabetologische Praxis, Bad Oeynhausen Herz-und Diabeteszentrum NRW, Bad Orb Spessart Klinik, Bad Reichenhall Kreisklinik Innere Med., Bad Salzungen Kinderklinik, Bautzen Oberlausitz KK, Bayreuth Innere Medizin, Berchtesgaden CJD, Berchtesgaden CJD-Beruf. REHA, Berlin DRK-Kliniken Pädiatrie, Berlin Lichtenberg – Kinderklinik, Berlin Virchow-Kinderklinik, Berlin Vivantes Hellersdorf Innere, Bielefeld Kinderarztpraxis, Bielefeld Kinderklinik Gilead, Bocholt Kinderklinik, Bochum Universitätskinderklinik St. Josef, Bodnegg - MVZ Wollmarshöhe, Bonn Uni-Kinderklinik, Braunschweig Kinderarztpraxis, Bremen - Kinderklinik Nord, Bremen Zentralkrankenhaus Kinderklinik, Bremerhaven Kinderklinik, Bruchweiler Edelsteinklinik Kinder-Reha, Böblingen Kinderklinik, Celle Kinderarztpraxis, Celle Klinik für Kinder- und Jugendmedizin, Chemnitz Kinderklinik, Coburg Innere Medizin, Coburg Kinderklinik, Coesfeld Kinderklinik, Darmstadt Kinderklinik Prinz. Margaret, Datteln Vestische Kinderklinik, Deggendorf Medizinische Klinik II, Deggendorf Pädiatrie-Praxis, Delmenhorst JHD Kinderklinik, Dessau Kinderklinik, Dessau amb. Kinderarztzentrum, Detmold Kinderklinik, Dortmund Johannes Hospital, Dortmund Kinderklinik, Dortmund Knappschaftskrankenhaus Innere, Dortmund Medizinische Kliniken Nord, Dortmund-St. Josefshospital Innere, Dortmund-West Innere, Dresden Neustadt Kinderklinik, Dresden Uni-Kinderklinik, Duisburg Homberg Helios Rhein-Ruhr Kliniken GmbH, Duisburg Sana Kinderklinik, Duisburg-St.Johannes Helios, Düren-Birkesdorf Kinderklinik, Düsseldorf Uni-Kinderklinik, Eckernförde Gem.-Prax, Erfurt Kinderklinik, Erlangen Uni-Kinderklinik, Essen Diabetes-SPP, Essen Diabetes-Schwerpunktpraxis, Essen Elisabeth Kinderklinik, Essen Kinderarztpraxis, Essen Uni-Kinderklinik, Esslingen Klinik für Kinder und Jugendliche, Esslingen Schwerpunktpraxis,

Filderstadt Kinderklinik, Flensburg Diakonissen Kinderklinik, Frankenthal Kinderarztpraxis, Frankfurt Diabeteszentrum Rhein-Main-Erwachsenendiabetologie, (Bürgerhospital), Frankfurt Diabeteszentrum Rhein-Main-pädiat. Diabetologie, (Clementine-Hospital), Frankfurt Uni-Kinderklinik, Frankfurt-Höchst, Städtische Kinderklinik, Frankfurt-Sachsenhausen Innere, Frankfurt-Sachsenhausen Innere MVZ, Freiburg St. Josef Kinderklinik, Freiburg Uni Innere, Freiburg Uni-Kinderklinik, Freudenstadt Kinderklinik, Fulda Kinderklinik, Fürth Kinderklinik, Gaissach Fachklinik der Deutschen Rentenversicherung Bayern Süd, Garmisch-Partenkirchen Kinderklinik, Garmisch-Partenkirchen Klinikum Pädiatrie, Gelnhausen Kinderklinik, Gelsenkirchen Kinderklinik Marienhospital, Gera Kinderklinik, Gießen Uni-Kinderklinik, Greifswald Uni-Kinderklinik, Gummersbach Oberbergklinikum, Görlitz Städtische Kinderklinik, Göttingen Uni-Kinderklinik, Hagen Kinderklinik, Halle Uni-Kinderklinik, Hamburg Altonaer Kinderklinik, Hamburg Kinderklinik Wilhelmstift, Hamburg-Nord Kinder-MVZ, Hameln Kinderklinik, Hamm Kinderklinik, Hanau Kinderklinik, Hanau diabetol. Schwerpunktpraxis, Hannover DM-SPP, Hannover Kinderklinik MHH, Hannover Kinderklinik auf der Bult, Haren Kinderarztpraxis, Heide Kinderklinik, Heidelberg St. Josefskrankenhaus, Heidelberg Uni-Kinderklinik, Heidenheim Kinderklinik, Heilbronn Kinderklinik, Herdecke Kinderklinik, Herford Kinderarztpraxis, Herford Klinikum Kinder & Jugendliche, Heringsdorf Inselklinik, Hildesheim Bernward Krks Kinderheilkunde, Hildesheim Kinderarztpraxis, Hildesheim Kinderklinik, Hof Kinderklinik, Hohenmölsen Diabeteszentrum, Homburg Uni-Kinderklinik Saarland, Idar Oberstein Schwerpunktpraxis, Itzehoe Kinderklinik, Jena Kinderarztpraxis, Jena Uni-Kinderklinik, Kaiserslautern Kinderarztpraxis, Kaiserslautern-Westpfalzklinikum Kinderklinik, Karlsburg Klinik für Diabetes & Stoffwechsel, Karlsruhe Schwerpunktpraxis, Karlsruhe Städtische Kinderklinik, Kassel Klinikum Kinder- und Jugendmedizin, Kaufbeuren Kinderklinik, Kempten Oberallgäu Kinderklinik, Kiel Städtische Kinderklinik, Kiel Universitäts-Kinderklinik, Kirchen DRK Krankenhaus Kinderklinik, Kirchheim-Nürtingen Innere, Koblenz Kinderklinik Kemperhof, Konstanz Kinderklinik, Krefeld Kinderklinik, Hannover DM-SPP, Hannover Kinderklinik MHH, Hannover Kinderklinik auf der Bult, Haren Kinderarztpraxis, Heide Kinderklinik, Heidelberg St. Josefskrankenhaus, Heidelberg Uni-Kinderklinik, Heidenheim

Kinderklinik, Heilbronn Kinderklinik, Herdecke Kinderklinik, Herford Kinderarztpraxis, Herford Klinikum Kinder & Jugendliche, Heringsdorf Inselklinik, Hildesheim Bernward Krks Kinderheilkunde, Hildesheim Kinderarztpraxis, Hildesheim Kinderklinik, Hof Kinderklinik, Hohenmölsen Diabeteszentrum, Homburg Uni-Kinderklinik Saarland, Idar Oberstein Schwerpunktpraxis, Itzehoe Kinderklinik, Jena Kinderarztpraxis, Jena Uni-Kinderklinik, Kaiserslautern Kinderarztpraxis, Kaiserslautern-Westpfalzklinikum Kinderklinik, Karlsburg Klinik für Diabetes & Stoffwechsel, Karlsruhe Schwerpunktpraxis, Karlsruhe Städtische Kinderklinik, Kassel Klinikum Kinder- und Jugendmedizin, Kaufbeuren Kinderklinik, Kempten Oberallgäu Kinderklinik, Kiel Städtische Kinderklinik, Kiel Universitäts-Kinderklinik, Kirchen DRK Krankenhaus Kinderklinik, Kirchheim-Nürtingen Innere, Koblenz Kinderklinik Kemperhof, Konstanz Kinderklinik, Krefeld Kinderklinik, München-Gauting Kinderarztzentrum, München-Schwabing Kinderklinik, Münster Herz Jesu Innere, Münster Ludgerus-Kliniken GmbH, Münster St. Franziskus Kinderklinik, Münster Uni-Kinderklinik, Neuburg Kinderklinik, Neunkirchen Gemeinschaftspraxis Kinderheilkunde, Neunkirchen Marienhausklinik Kohlhof Kinderklinik, Neuss Lukas-Krankenhaus Kinderklinik, Neuwied Kinderklinik Elisabeth, Nürnberg Cnopfsche Kinderklinik, Nürnberg Uniklinik Zentrum f Neugeb./Kinder & Jugendl., Oberhausen Kinderklinik, Oberhausen Kinderpraxis, Oberhausen St.Clemens Hospitale Sterkrade, Offenburg Kinderklinik, Oldenburg Kinderklinik, Oldenburg Schwerpunktpraxis Pädiatrie, Olpe pädiatrische Gemeinschaftspraxis, Osnabrück Christliches Kinderhospital, Paderborn St. Vincenz Kinderklinik, Passau Kinderklinik, Pforzheim Kinderklinik, Pirmasens Städtisches Krankenhaus Innere, Plauen Vogtlandklinikum, Ravensburg Kinderklink St. Nikolaus, Regensburg Kinderklinik St. Hedwig, Remscheid Kinderklinik, Rendsburg Kinderklinik, Reutlingen Kinderarztpraxis, Reutlingen Kinderklinik, Rheine Mathiasspital Kinderklinik, Rosenheim Kinderklinik, Rosenheim Schwerpunktpraxis, Rostock Uni-Kinderklinik, Rotenburg/Wümme Agaplesion Diakonieklinikum Kinderabteilung, Rottweil Gemeinschaftspraxis für Innere Medizin, Rüsselsheim Kinderklinik, Rüsselsheim MVZ, Saaldorf-Surheim Diabetespraxis, Saarbrücken Kinderklinik Winterberg, Scheidegg Prinzregent Luitpold, Schleswig Heliosklinik Kinderklinik, Schw. Gmünd Stauferklinik

Kinderklinik, Schweinfurt Kinderklinik, Schwerin Kinderklinik, Schwäbisch Hall Diakonie Kinderklinik, Siegen Kinderklinik, Singen Hegau Bodensee-Klinikum Kinderklinik, Singen Kinderarztpraxis, Speyer Diakonissen Stiftungskrankenhaus Pädiatrie, St. Augustin Kinderklinik, Stade Kinderklinik, Stolberg Kinderklinik, Stuttgart Olgahospital Kinderklinik, Suhl Kinderklinik, Sylt Rehaklinik, Tettnang Innere Medizin, Traunstein Kinderklinik, Trier Kinderklinik der Borromäerinnen, Tübingen Uni-Kinderklinik, Ulm Endokrinologikum Amedes, Ulm Uni-Kinderklinik, Vechta Kinderklinik, Viersen Kinderkrankenhaus St. Nikolaus, Viersen internist. Praxis, Villingen-Schwenningen Schwarzwald Baar Klinikum Kinderklinik, Volkertshausen Gemeinschaftspraxis, Waldshut Kinderpraxis, Waren-Müritz Kinderklinik, Weiden Kinderklinik, Weingarten Kinderarztpraxis, Wesel Marienhospital Kinderklinik, Wiesbaden Helios Horst-Schmidt-Kinderkliniken, Wiesbaden Kinderklinik DKD, Wilhelmshaven Kinderarztpraxis, Wilhelmshaven Klinikum Kinderklinik, Winnenden Rems-Murr Kinderklinik, Witten Kinderarztpraxis, Wittenberg Kinderklinik, Worms - Weierhof, Worms Kinderklinik, Wuppertal Universitäts-Kinderklinik, Zweibrücken Kinderarztpraxis.
