# Supplementary material for: Metabolic control during the first two years of the COVID-19 pandemic in pediatric patients with type 1 diabetes: results from the German DPV initiative
Source: Acta Diabetol. 2023 Mar 4;60(6):757–66. doi: 10.1007/s00592-023-02050-x (PMC9985474; doi:10.1007/s00592-023-02050-x)

**Figure S1**

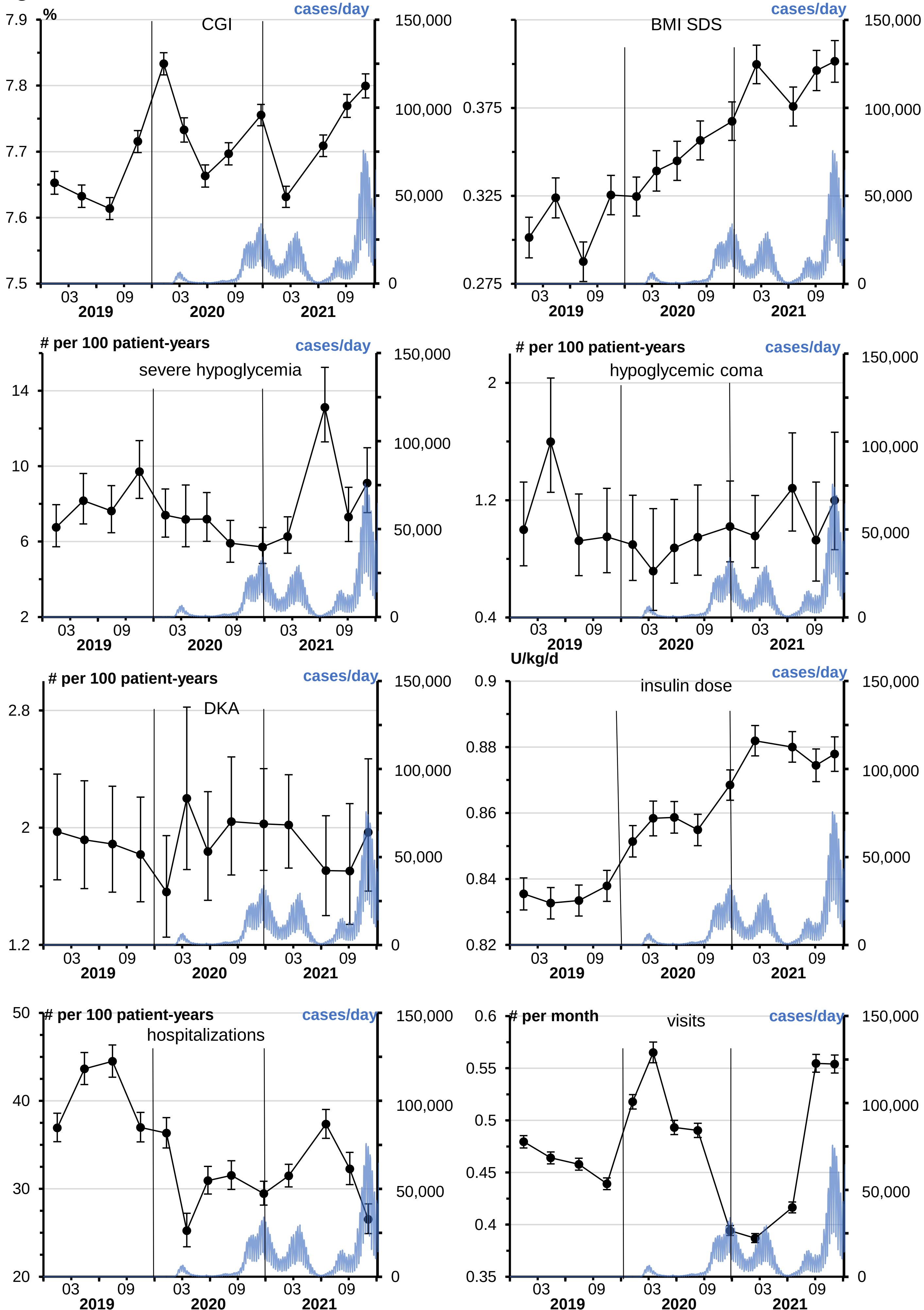

%
CGI
cases/day
7.9
7.8
7.7
7.6
7.5
150,000
100,000
50,000
0
03
09
2019
2020
2021
BMI SDS
0.375
0.325
0.275
# per 100 patient-years
severe hypoglycemia
14
10
6
2
hypoglycemic coma
2
1.2
0.4
U/kg/d
insulin dose
0.9
0.88
0.86
0.84
0.82
DKA
2.8
1.2
hospitalizations
50
40
30
20
# per month
visits
0.6
0.55
0.5
0.45
0.4
0.35

## Figure S2

### CGI

**All patients**

**Sensitivity analysis:**
**additional adjustment of models for**
**social deprivation index**

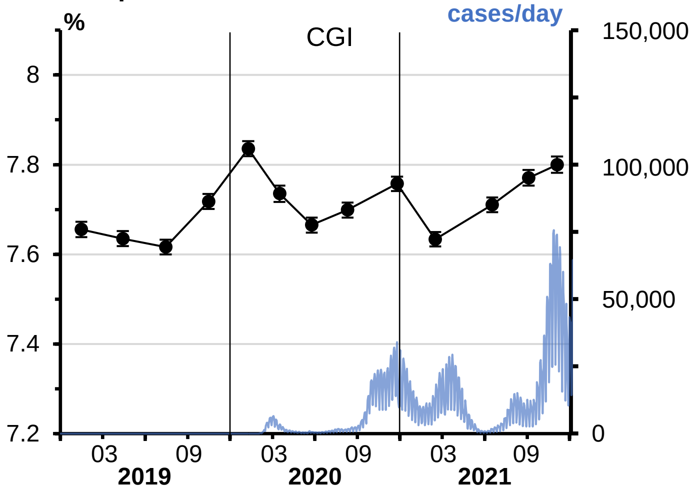


### BMI-SDS

**All patients**

**Sensitivity analysis:**
**additional adjustment of models for**
**social deprivation index**

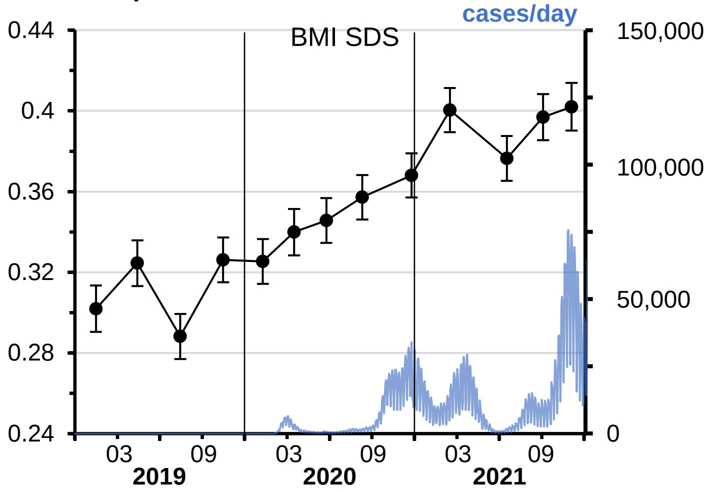


**Patients aged < 12 years**

**Sensitivity analysis:**
**additional adjustment of models for**
**social deprivation index**

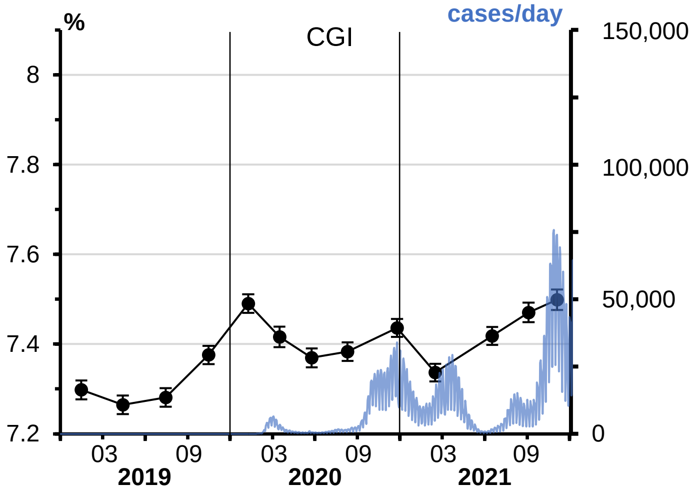


**Patients aged < 12 years**

**Sensitivity analysis:**
**additional adjustment of models for**
**social deprivation index**

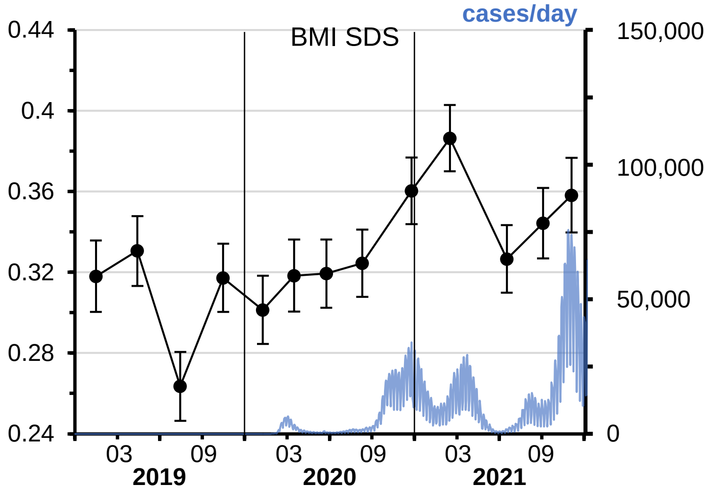


**Patients aged ≥ 12 years**

**Sensitivity analysis:**
**additional adjustment of models for**
**social deprivation index**

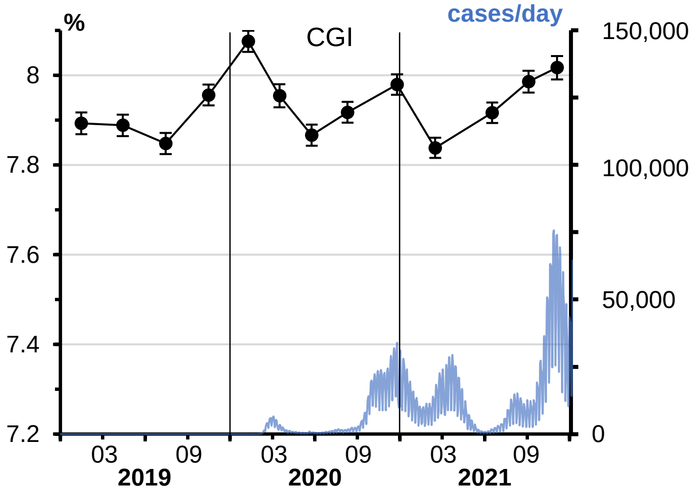


**Patients aged ≥ 12 years**

**Sensitivity analysis:**
**additional adjustment of models for**
**social deprivation index**

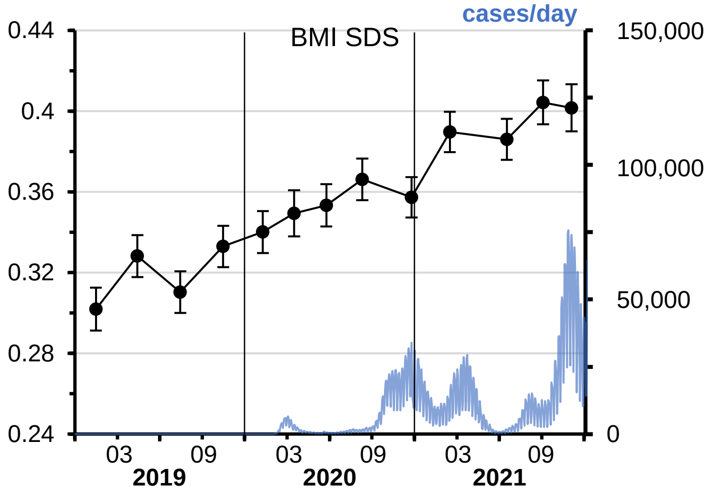

# Figure S3

**Stratified analysis**

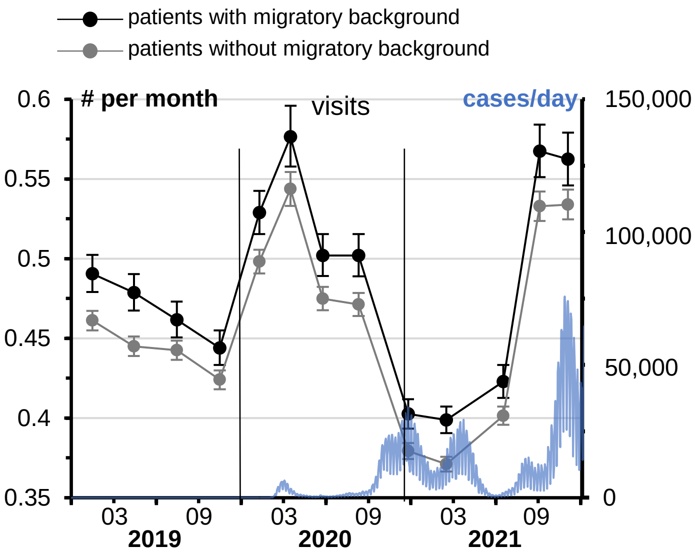


# Figure S4

**patients without CGM usage; laboratory-measured HbA1c**

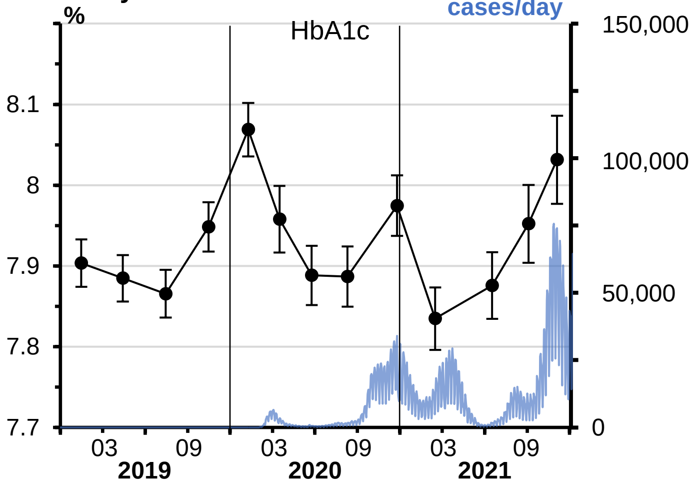


**patients without available time-in-range in the respective time-period laboratory-measured HbA1c**

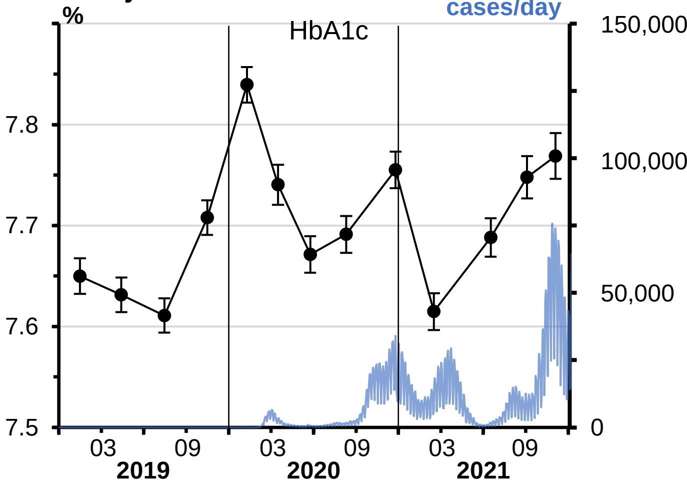

Supplement: Supplementary file 2 — Supplementary file2 (PDF 197 KB) [file 592_2023_2050_MOESM2_ESM.pdf]
